# Effect of Adding Kinesio Taping to Exercise Therapy in the Treatment of Patellofemoral Pain Syndrome

**DOI:** 10.3390/medicina59040754

**Published:** 2023-04-12

**Authors:** Jin Hyuck Lee, Hye Chang Rhim, Ki-Mo Jang

**Affiliations:** 1 Department of Sports Medical Center, Anam Hospital, Korea University College of Medicine, Seoul 02841, Republic of Korea; gnkfccc@hanmail.net; 2 Department of Physical Medicine and Rehabilitation, Harvard Medical School/Spaulding Rehabilitation Hospital, Boston, MA 02115, USA; hrhim@mgh.harvard.edu; 3 Department of Orthopaedic Surgery, Anam Hospital, Korea University College of Medicine, Seoul 02841, Republic of Korea

**Keywords:** patellofemoral pain syndrome, patellar taping, kinesio taping, quadriceps strength, acceleration time, patient-reported outcome

## Abstract

*Background and objectives:* Patellar taping has been widely used for the primary or adjunctive treatment of patellofemoral pain syndrome (PFPS); however, there are limited data in terms of functional outcomes. This study aimed to investigate whether there is any beneficial effect of adding Kinesio Taping^®^ (KT) to exercise therapy in the treatment of PFPS. *Materials and Methods:* Twenty patients (27.5 ± 5.4 years) with PFPS who applied KT and 19 patients (27.3 ± 7.4 years) who did not were included in this study. Quadriceps muscle strength and acceleration time (AT) were assessed using an isokinetic device. Patient-reported outcomes were evaluated using the Kujala anterior knee pain scale (AKPS). Both groups underwent one-month exercise therapy. *Results:* There was no significant difference in quadriceps strength, AT, and AKPS at baseline and at 1 month between the taping and non-taping groups (*p* > 0.05). However, for quadriceps muscle strength, the effect of time*group interaction was statistically significant (F(1.37) = 4.543, *p* < 0.05, partial eta squared 0.109), showing that improvement in the quadriceps strength was higher in the non-taping group than that in the taping group. *Conclusions:* Adding KT to exercise therapy did not elicit extra benefits in quadriceps muscle strength and AT, and AKPS among PFPS with abnormal patellar tracking at one month.

## 1. Introduction

Patellofemoral pain syndrome (PFPS) is one of the most commonly diagnosed conditions among adolescents and adults with knee complaints, accounting for approximately 25% of knee disorders diagnosed in sports medical clinics [1]. While the etiology of PFPS is suggested to be multifactorial, several contributing factors such as lower knee extension strength, quadriceps imbalance, quadriceps angle, decreased hip muscle strength, and overuse have been identified [2,3]. Therefore, physical therapy including strengthening and stretching exercises for hip and quadriceps muscles have been integral part of treating PFPS.

At the same time, other modalities such as patellar brace or taping have been used adjunctively with the goal of reducing pain and restoring proper patellar alignment. Two commonly used taping techniques for PFPS include Kinesio Taping^®^ Method (KT) and McConnell Taping Technique (MT). The MT uses rigid and highly adhesive tape that is structurally supportive while the KT uses more compliantly adhesive taping that can stretch to 130–140% of its original length allowing for enough range of motion [4,5]. MT is thought to correct patellar malalignment and facilitate vastus medialis oblique (VMO) contractions, thereby reducing pain [6]. On the other hand, KT is theorized to increase proprioception, improve vascular flow, [7] and help restore proper joint alignment [5]. Due to its low cost and wide availability, patellar taping has been frequently applied in clinical practice either as a sole treatment or as part of a treatment program.

Up to date, many studies have explored the effects of patellar taping in function and pain among PFPS patients. One of the most recent systematic reviews in fact suggested that patellar taping should be used only as a supplement to traditional exercise treatment for PFPS rather than used as a sole treatment [8]. However, this review was based on only five studies mixed with MT and KT and focused only on improvement in visual analog scale for pain. Other researches have investigated the effects of patellar taping on functional performance, [9,10] quadriceps muscle strength, [11,12,13] and muscle activation [14,15]. Still, the evidence is conflicting, and generalization of previous studies is limited by the heterogeneity of treatment duration and concurrent intervention [16]. Since there is no consensus regarding the best approach for the treatment of PFPS, an ongoing research effort needs to be made.

Considering such gap in literature, the purpose of this study was to analyze the additional value of KT among PFPS patients with abnormal patellar tracking who were concurrently treated with exercise therapy in terms of functional outcomes including quadriceps muscle strength and rate of force generation, and patient-reported outcomes including Kujala anterior knee pain scale (AKPS). We hypothesized that KT may have additional benefits in terms of functional and patient-reported outcomes.

## 2. Materials and Methods

### 2.1. Study Design and Patient Enrollment

**Figure 1:**
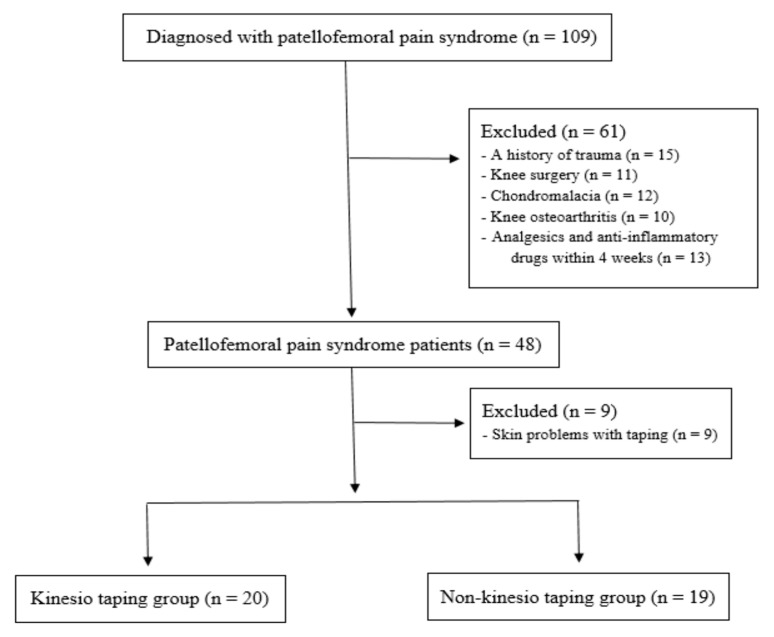
Study flow diagram.

This study is a retrospective comparative study and followed the Strengthening the Reporting of Observational studies in Epidemiology (STROBE) guidelines for non-pharmacological treatment, and all patients and/or their legal guardians provided informed consent. The protocol of this research was approved by the institutional review board at our institution (2017AN0178). A total of 109 patients diagnosed with PFPS by an orthopedic surgeon were retrospectively reviewed. For the diagnosis of PFPS, it is necessary for the patients to present retropatellar or anterior aspect knee pain during at least two of the following daily activities: running, sitting for long hours, walking up and down stairs, kneeling, crouching or jumping. Plain radiographs were used to assess patello-femoral structural abnormalities, such as patellar tilting, subluxation, or trochlear groove [17,18]. If the patients presented anterior knee pain with a catching or click around the patella during knee flexion-extension, they were considered to have abnormal tracking of the patella. The exclusion criteria were: (1) history of trauma around the knee joint, knee surgery, knee osteoarthritis or chondromalacia; (2) taking anti-inflammatory medications or analgesics within 4 weeks; and (3) KT-related allergic reaction on skin. The selection led to the enrollment of 39 patients who were included in this study (Figure 1). Twenty patients were included in the taping group and the remaining 19 patients in the non-taping group. Table 1 shows the demographic and baseline data of the enrolled patients. There were no significant differences between the taping and non-taping groups regarding sex, age, height, weight, body mass index, steps, injured side, and sports and activity level (*p* > 0.05).

### 2.2. Evaluation Tests

#### 2.2.1. Quadriceps Muscle Strength and Rate of Force Generation

Quadriceps muscle strength and rate of force generation were measured using an isokinetic device (Biodex multi-joint system 4, Biodex Medical Systems, Inc., Shirley, NY, USA) [19,20]. The lateral femoral condyle of the knee joint was carefully aligned with the rotational axis of the isokinetic dynamometer. The patients were asked to perform five maximal repetitions of knee flexion-extension motions at 180°/s. Normalization of the peak torque by body weight (peak torque/body weight, N·m·kg^−1^ × 100) was used to assess muscle strength. A fast coordination of muscle reaction is critical to protect joints, [21] and the muscle reaction mechanism is closely associated with the arthrokinetic reflex, which is influenced by velocity and acceleration [20,22]. Therefore, rate of force generation in quadriceps muscle was measured according to the acceleration time (AT, milliseconds) in the isokinetic muscle testing. AT was defined as the time it took up to the preset-angular velocity (180°/s in the current study) during maximal muscle contraction [20,22]. Thus, slow AT indicated poor force generation rate. Isokinetic muscle tests were carried out at baseline and one month after therapeutic exercise and patellar taping.

#### 2.2.2. Patient-Reported Outcomes: Pain and Function

The AKPS was used to evaluate function and pain. The AKPS consists of 13 items and is graded on a scale of 0 to 100, with 100 being the highest possible score. Higher AKPS scores mean lower functional limitation. Based on a previous study, [23] changes in AKPS by 8–10 points in PFPS patients were considered as minimal clinically important difference (MCID).

### 2.3. Patellar Taping Intervention

**Figure 2:**
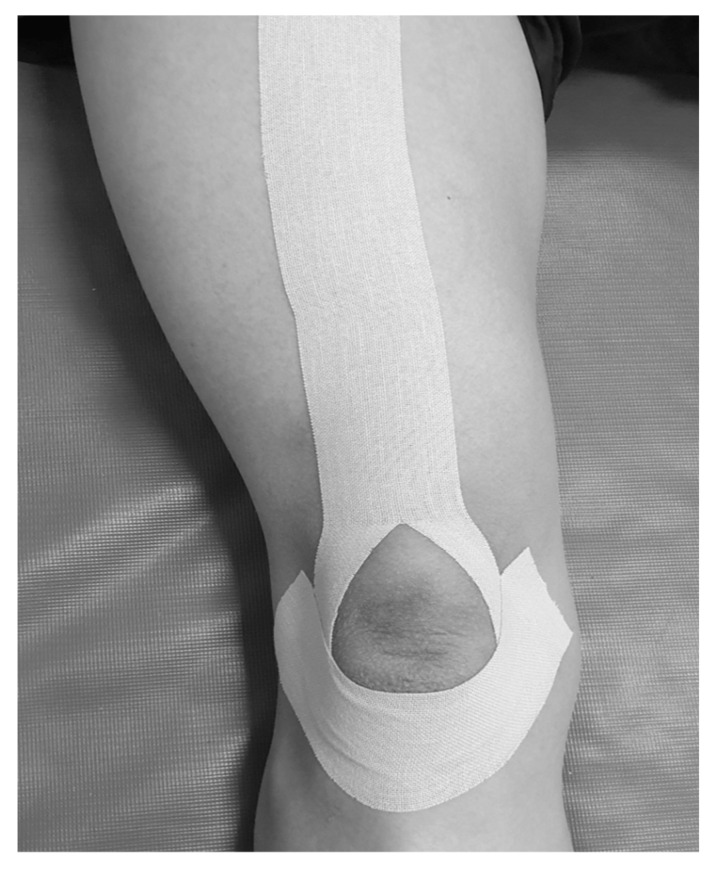
Kinesio taping technique.

KT with a thickness of 0.5 mm and a width of 5 cm (Nitto Denko Top^®^, Osaka, Japan) was utilized for patellar taping, and the taping technique was adopted from a previous study [8]. The taping was wrapped around the patella along the quadriceps tendon and muscle to protect and stabilize the patella (Figure 2). The professionally trained physical therapists applied the initial KT in the first session of initiating exercise therapy. Then, the patients were educated on the taping technique to apply the taping by themselves while performing daily activities and exercise. The patients were taught to apply the KT every day from morning to evening for a month.

### 2.4. Conservative Therapeutic Exercise

The protocol of therapeutic exercise was adopted from a previous study by our research group, [19] and the therapeutic exercise was carried out in both the taping and non-taping groups for a month. The only difference between the two groups was whether or not the taping was applied. All the patients visited our sports medicine center for one-hour training session twice a week. They were instructed to perform open kinetic-chain exercises, including multi-directional straight leg raise and knee extension with knee adduction in the pain-free range to strengthen the quadriceps and hip muscles. In addition, they were taught to perform closed kinetic-chain exercises, including wall squat exercise with knee adduction and single-leg squat exercise in 50° below. Both single-leg balance exercises were carried out to enhance neuromuscular control and proprioception. The hip and core muscle strengthening regimen was also performed. All patients were instructed to carry out the exercises at home twice a day. Compliance to the home exercise therapy was monitored by the physical therapists.

### 2.5. Statistical Analysis

Based on previous studies regarding quadriceps muscle strength in patients with PFPS, [11,24] clinically significant difference was considered quadriceps muscle strength more than at least 10%. In a pilot study with 5 knees in each group to assess a between-group difference in quadriceps muscle strength of >10%, more than 38 patients were needed (Cohen’s d effect size: 0.959). Therefore, in the current study, 20 patients in the taping group and 19 patients in the non-taping group were enrolled. The power of this study was 0.820 to detect a significant difference in quadriceps muscle strength of >10%. The Fisher’s test and independent t-test were used to compare the demographic and baseline characteristics of the patients. We used the paired t-test to evaluate any significant change in muscle strength and AT of quadriceps, and AKPS between pre- and post-intervention in each group. The two-way repeated measures analysis of variance (ANOVA) was used to compare whether the mean change in the outcome measures from pre- to post-intervention differed in the two groups. The Shapiro-Wilk test was performed to assess the normality distribution of the continuous variables. We performed the Mauchly’s test of sphericity and the Box’s test of equality of covariance matrices to assess whether the assumptions for ANOVA were met. The collected data was analyzed using SPSS Statistics version 21.0. (IBM Corp, Armonk, NY, USA), and *p*-values < 0.05 were considered statistically significant.

## 3. Results

### 3.1. Comparison of Functional Outcomes in Each Group

In the taping group, quadriceps muscle strength (87.3 ± 31.3 N·m·kg^−1^ × 100 vs. 137.8 ± 52.2 N·m·kg^−1^ × 100, effect size (Cohen’s d) = −1.17), quadriceps AT (60.4 ± 16.1 ms vs. 48.7 ± 14.8 ms, effect size = 0.75), and AKPS (52.5 ± 7.9 points vs. 68.3 ± 9.3 points, effect size = −1.83), statistically significantly improved at a month after intervention (Table 2).

In the non-taping group, quadriceps muscle strength (88.9 ± 37.8 N·m·kg^−1^ × 100 vs. 162.9 ± 28.5 N·m·kg^−1^ × 100, effect size = −2.21), quadriceps AT (70.5 ± 18.9 ms vs. 54.2 ± 13.0 ms, effect size = 1.00), AKPS (49.7 ± 6.6 points vs. 69.9 ± 7.4 points, effect size = −2.88), statistically significantly improved at a month after intervention (Table 2).

### 3.2. Comparison of Functional Outcomes between Two Groups

No statistically significant difference was found in terms of quadriceps muscle strength at baseline and at 1 month between the two groups (Table 2). However, the effect of time*group interaction was statistically significant (F (1.37) = 4.543, *p* < 0.05, partial eta squared 0.109) for quadriceps muscle strength, indicating that improvement in the quadriceps muscle strength was significantly higher in the non-taping group at 1 month than that in the taping group.

No significant differences were identified in terms of quadriceps muscle AT and AKPS between the two groups at baseline and at 1 month (Table 2). The effects of time*group interactions for quadriceps AT (F (1,37) = 1.046, *p* = 0.313, partial eta squared 0.028) and AKPS (F (1.37) = 1.485, *p* = 0.231, partial eta squared 0.039) were not statistically significant.

## 4. Discussion

The purpose of our study was to investigate whether the KT application would elicit more benefits in quadriceps muscle strength, AT of quadriceps, and patient-reported functional outcomes than undergoing exercise therapy alone. While all outcomes improved in both groups at one month, unlike our hypothesis, quadriceps AT, and AKPS did not significantly differ between the two groups. Unexpected and novel finding of this study was that the improvement of quadriceps muscle strength was greater in the non-taping group than the taping group. 

### 4.1. Effect of Adding KT on Quadriceps Muscle Strength

Several studies have investigated the effect of patellar taping on quadriceps strength, and the results were conflicting [11,12,13,25,26,27,28]. Most of the studies that showed potential benefits of patellar taping in quadriceps strength, in fact, tested for immediate effect of patellar taping [11,13]. Furthermore, no significant difference in quadricep strength was observed when there was placebo taping group (Keet et al. used placebo tape that did not have medial glide component while Aytar et al. used placebo tape that did not have stretching property) [12,13]. Therefore, for those studies that compared taping with no taping, [11] this immediate benefit could have been a placebo effect. Also, whether this immediate effect would sustain for a longer period is questionable from those studies. Kaya et al. evaluated patients after three months of applying short-duration patellar taping during exercise program and found that the combination of exercise and taping can improve knee extensor muscle strength. However, their main limitation was that there was no group with exercise therapy alone without taping [26]. As a result, this study could not demonstrate whether the improvement of knee extensor muscles derived from exercise program or taping. Our result was most consistent with the findings from Kowall et al. which showed that while exercise therapy improved quadriceps strength, there was no beneficial effect of adding a patellar taping to exercise therapy [25]. In addition, Mason et al. found that both strengthening and stretching groups resulted in increased quadriceps strength, but taping only group failed to show improvement [27]. Eventually, these findings indicate that the improvement in quadriceps muscle strength over time in PFPS patients might be attributed to therapeutic exercises rather than taping itself.

Unexpected finding from our study was that quadriceps strength improved in greater degree in the non-taping group than the taping group. A possible mechanism for this finding may be inhibitory effect of taping. Alexander et al. demonstrated an inhibitory effect of taping in gastrocnemius and trapezius muscles [29]. It is postulated that inhibition of muscles can be accomplished by stretching the Golgi tendon organ at the distal end of the muscle. Willy et al. reported that patellar taping is not recommended for the aim of improving muscle strength in patients with PFPS [3]. Therefore, further studies would be necessary to confirm whether chronic use of taping may interfere with positive effects of exercise therapy in terms of quadriceps strength among PFPS patients.

### 4.2. Effect of Additional KT on the Rate of Force Generation in Quadriceps Muscle

Previous studies reported that patellar taping may change the quadriceps muscle activation in patients with PFPS [14,15,30]. However, we failed to show significant difference on AT of quadriceps between the two groups in this study. A possible explanation may derive from differences in muscle evaluation methods. In our study, an isokinetic device was used to measure quadriceps AT. However, in some previous studies, [14,15,30] electromyography (EMG) was used to assess VMO and vastus lateralis (VL), which are the quadriceps muscle components. Gilleard et al. [14] and Cowan et al. [15] found that the VMO was earlier activated than the VL after taping while Crhistou et al. reported increased VMO activity and decreased VL activity with taping in PFPS patients. Unfortunately, the methodology of the current study did not allow the measurement of AT in each component of the vasti muscles, and based on the previous studies, evaluating VMO and VL individually may be more important. Nonetheless, it could also be possible that therapeutic exercises might have played a greater role in improving quadriceps AT. Irish et al. [31] demonstrated that VMO muscle activation can be improved by certain closed-kinetic chain and hip adduction exercises, which were in fact core part of therapeutic exercises prescribed to both groups in our study. Given that there was no difference between the groups at one month, the application of patellar taping did not provide additional benefits in terms of the force generation rate in quadriceps muscle.

### 4.3. Effect of Additional KT on Patient-Reported Pain and Functional Performance (AKPS)

In the present study, while both groups demonstrated significant improvement AKPS, there was no significant difference between the taping and non-taping groups at one month. This result contradicts the most recent systematic review that concluded there is evidence for superior decrease in pain when taping is combined with exercise compared to exercise alone among PFPS patients [8]. However, this systematic review only included five studies, three of which used MT. Other studies that used KT did not show any superior results with taping additional to exercise in terms of pain and functional performance at 6 weeks [6,9,10] and at 12 weeks [10]. Consistent with these studies, our study found that KT did not elicit additional benefit in pain and functional performance. In light of significant improvement in quadriceps strength and AT in both groups, the reduction of pain and improvement of functional performance may be attributed to strength gains and faster force generation rate rather than KT’s purported function of pain reduction [32].

### 4.4. Clinical Implications

Despite high prevalence of PFPS and its persistence and recurrence, there is no consensus on the best treatment approach. The most recent systematic review with network meta-analysis on comparative effectiveness of treatments for PFPS concluded that there was insufficient evidence to suggest a specific type of physical treatment over another while any type of physical treatments including exercise, orthoses, or patellar taping is better than wait-and-see approach [33].

In contrast, according to the recent clinical practice guidelines for patellofemoral pain, [3] patellar taping may be effective in terms of immediate pain reduction and improved outcomes of exercise therapy in four weeks. Our study confirms that exercise therapy including open and close kinetic chain exercises and targeting proprioception and neuromuscular control should be the core component of treating PFPS. Also, our finding showed that adding KT to the exercise therapy does not bring extra benefits in terms of quadriceps muscle strength and AT, pain, and functional performance. Rather, our study is first to suggest that the application of KT for 4weeks may interfere with quadriceps strength gain.

### 4.5. Limitations

This study has several limitations. First, our study was a retrospective study which is prone to selection bias. Hence, unlike some prospective studies which implemented placebo taping groups, we could not have a placebo taping group due to the retrospective nature of our study. Further prospective, randomized controlled trials implementing placebo taping are needed to confirm whether the application of KT interferes with quadriceps strength gain and whether there is any benefit over placebo effect. Second, we did not evaluate psychological factors such as kinesiophobia. In a systematic review [34] and a recent study [35], authors demonstrated that psychological factors (kinesiophobia and fear-avoidance) were related to pain and physical function in PFPS patients. In particular, a recent study reported that kinesiophobia improved immediately after two weeks with application of the soft knee brace in patient with PFPS [36]. Hence, evaluation of psychological factors may be needed to clarify our findings on the effectiveness of taping in patients with PFPS. Third, we did not confirm whether KT could alleviate abnormal patellar tracking during muscle performance tests using an isokinetic device. Fourth, we could not measure each individual vastus muscle (i.e., VMO or VL) reaction time which could be more clinically relevant in the setting of PFPS. Finally, this was a relatively short-term follow-up study. 

## 5. Conclusions

Adding KT to exercise therapy did not elicit extra benefits in quadriceps muscle strength and AT, and AKPS among PFPS with abnormal patellar tracking at one month. There is a possibility that KT might rather interfere with quadriceps strength gain over a period of one month. Clinicians and physical therapists should be aware of conflicting and low scientific evidence of patellar taping in the treatment of PFPS and disclose this information to patients interested in taping since taping has been and will continue to be used due to wide availability, low cost, and anecdotal benefits.

## Figures and Tables

**Table 1:** Demographic data of enrolled patients.

	Taping Group (*n* = 20)	Non-Taping Group (*n* = 19)	*p*-Value
Sex (male/female)	5/15	3/16	0.305
Age (years)	27.5 ± 5.4	27.3 ± 7.4	0.950
Height (cm)	167.5 ± 6.3	166.5 ± 7.8	0.658
Weight (kg)	62.6 ± 10.2	61.9 ± 6.9	0.804
Body mass index (kg/m^2^)	21.9 ± 2.3	22.2 ± 1.7	0.634
Steps/day	7829 ± 1511.7	8001 ± 1342.3	0.461
Injured side (right/left)	15/5	10/9	
Sports and activity, *n* (low:high)	9/11	7/12	

Note: The values are expressed as mean ± standard deviation.

**Table 2:** Functional outcomes comparison between the groups.

		Taping Group	Non-Taping Group	MD (95% CI)	Cohen’s d	*p* Value
Quadriceps strength	Pretest	87.3 ± 31.3	88.9 ± 37.8	−1.6 (−23.4 to 20.2)	−0.04	0.884
	Posttest	137.8 ± 52.2	162.9 ± 28.5	−25.1 (−52.6 to 2.4)	−0.59	0.072
	MD (95% CI)	−50.5 (−65.8 to −35.15)	−74.0 (−91.5 to −56.6)			
	Cohen’s d	−1.17	−2.21			
	*p* value	0.001	0.001			
Quadriceps AT	Pretest	60.4 ± 16.1	70.5 ± 18.9	−10.1 (−21.6 to 1.3)	−0.57	0.081
	Posttest	48.7 ± 14.8	54.2 ± 13.0	−5.5 (−14.6 to 3.6)	−0.39	0.227
	MD (95% CI)	11.7 (5.3 to 18.0)	16.3 (9.3 to 23.4)			
	Cohen’s d	0.75	1.00			
	*p* value	0.001	0.001			
AKPS	Pretest	52.5 ± 7.9	49.7 ± 6.6	2.7 (−1.9 to 7.5)	0.38	0.244
	Posttest	68.3 ± 9.3	69.9 ± 7.4	−1.6 (−5.5 to 5.4)	−0.19	0.986
	MD (95% CI)	−15.8 (−21.2 to −13.6)	−20.2 (−23.2 to −17.3)			
	Cohen’s d	−1.83	−2.88			
	*p* value	0.001	0.001			

MD, mean difference; CI, confidence interval; AT, acceleration time; AKPS, anterior knee pain scale. The measurement unit of quadriceps strength was N·m·kg^−1^ × 100 The measurement unit of quadriceps AT was milliseconds. The measurement unit of AKPS was points. Values are expressed as mean ± standard deviation.

## Data Availability

The data presented in this study are available on request from the corresponding author.
